# Flexible PVDF membranes with exceptional robust superwetting surface for continuous separation of oil/water emulsions

**DOI:** 10.1038/s41598-017-14429-2

**Published:** 2017-10-26

**Authors:** Zhu Xiong, Haibo Lin, Fu Liu, Peng Xiao, Ziyang Wu, Tiantian Li, Dehong Li

**Affiliations:** Polymer and Composite Division, Ningbo Institute of Material Technology & Engineering, Chinese Academy of Sciences, Ningbo, China

## Abstract

Instability of superwetting surface is the stumbling block of flexible polymeric membranes for continuous separation of water-in-oil or oil-in-water emulsions. Manipulation of rigid superwetting nano-TiO_2_ on hierarchical poly(vinylidene fluoride) (PVDF) membrane by mimicking the plant roots holding soil behaviour enabled the generation of robust superwetting surface withstanding the harshly physical and chemical torture. The unique interface combination, which fabricated by a compacted nano-layer with the thickness of ~20 μm, was disclosed by systematic structure characterization. As demonstrated by SEM, LSCM and nano-CT, the pristine PVDF membrane with large quantities of cilia-like micro/nano-fibrils can function as the plant roots to capture, cage and confine the nanoparticles to form a robustly rigid nano-coating. The as-prepared membranes showed excellent durable separation performance both in varieties of stabilized water-in-oil and oil-in-water emulsion separation for a long term with few nanoparticles loss in a continuous crossflow mode. The strategy of assembling rigid inorganic nano-particles on flexible surface offers a window of opportunity for preparation of robust organic-inorganic hybrid membranes not only for continuous oil/water emulsion separation, but also for other functional application, such as electric conduction, heat conduction, ion exchange, and in membrane catalytic reactors etc.

## Introduction

The globally increasing oil pollution related with petrochemical, textile, and machining industries as well as the frequent oil spill accidents, has raised much concern about the potential negative effects on aquatic ecosystems and human health^1–3^. Facing these tough challenges, scientists have devoted themselves to developing superwetting materials for high efficient oil/water separation. Among them, membrane has been acknowledged as one of most advanced technologies for the separation of stabilized water/oil micro-emulsions due to the confined pore size^4,5^, comparing with traditional techniques^6,7^. Very recently, a great quantity of works have been conducted to design hierarchical micro-/nano-structured interface to acquire special super-wettability on polymeric membranes for oil/water emulsion separation, most of which are focusing on improving the selectivity and permeability^1,4,8–13^.

Nevertheless, the excellent separation performances of aforementioned membranes are usually achieved with simulated emulsions in an uncontinuous mode^9–13^. Usually, the uncontinuous mode is operated by a dead-end filter, which is difficult to meet the actual demand. In order to continuously treat the wastewater, the membrane is generally designed as a crossflow model in the industry. However, in the preparation and application of the membrane device, the membrane usually associated with unexpected abrasion, hydraulic pressure, chemical corrosion, high or low temperature torture. Especially for the superwetting membranes, their surface hierarchical structure will be seriously crashed under the above discussed damages due to the inherent creep deformation of polymers^14^, which results in the irreversible malfunction of superwetting behavior^15^.

Recently, the polymeric substances with the coating of fluorinated alkyl silane (FAS) modified silica or titanium dioxide nanoparticles showed remarkable durability against strong acid, strong alkali, repeated machine washes, boiling water and severe abrasion damages, whilst retaining its superhydrophobicity^16,17^. Thus, integrating the rigid inorganic nanoparticles with flexible polymeric membranes provided a pathway to solve the instability of superwettabilty. Thus, membranes decorated with TiO_2_^18^, SiO_2_^19^, CaCO_3_^20^, graphene^21^, and carbon nanotube^22^, which aimed to obtain the efficiently superwetting membranes, were widely investigated. Unfortunately, in their results, you will find that almost all oil/water separation efficiency and flux of these membranes were obtained by a dead-end filter. However, the dead-end filter could only evaluate the oil/water separation function of supperwetting membrane in a short term or in an ideal state. Just as we discussed above, membranes should operate in a crossflow model for the continuous separation. Considering that, we should resolve the harsh physical and chemical damages on the superwetting stability of membrane surface for the practical application requirement. Besides, the contacting cross flowed fluid would also generate a persistent crossflow shear force, and then gradually washed out the coated nano-particles from the membrane surfaces while launched the continuous separation for a long time. This is like the phenomenon of “constant dropping wears the stone”. As a result, the superwetting membrane will lost its efficiency on the separation of oil/water emulsion after a long term operation. Thus, the superwetting stability of inorganic nanoparticles immobilized on the flexible membrane surface under harsh oil/water separation environment remains the biggest challenge.

In fact, there was no literature reported that the oil/water emulsion separation could be successfully finished by the membrane via a crossflow filtration system due to the instability of superwetting surface. Therefore, micro-/nano- structure stability of polymeric membrane surface is crucial to accomplish the continuous separation of stabilized emulsions, especially for the inorganic materials decorated membranes. In nature, it is interestingly seen that the plant roots with macro-multiscale structure can robustly immobilize the soil particles even in mountain, river and desert^23,24^. Besides, a plenty of mobile sand, dust and other micro-particles could be gradually captured by the vast plant roots, and then became difficult to remove. Inspired by that, membranes with root-like micro/nano-structures might have the similar ability to immobilize the super-wettable nano-particles on their surface as the ideal model for oil/water emulsion separation or other functional implication.

**Figure 1:**
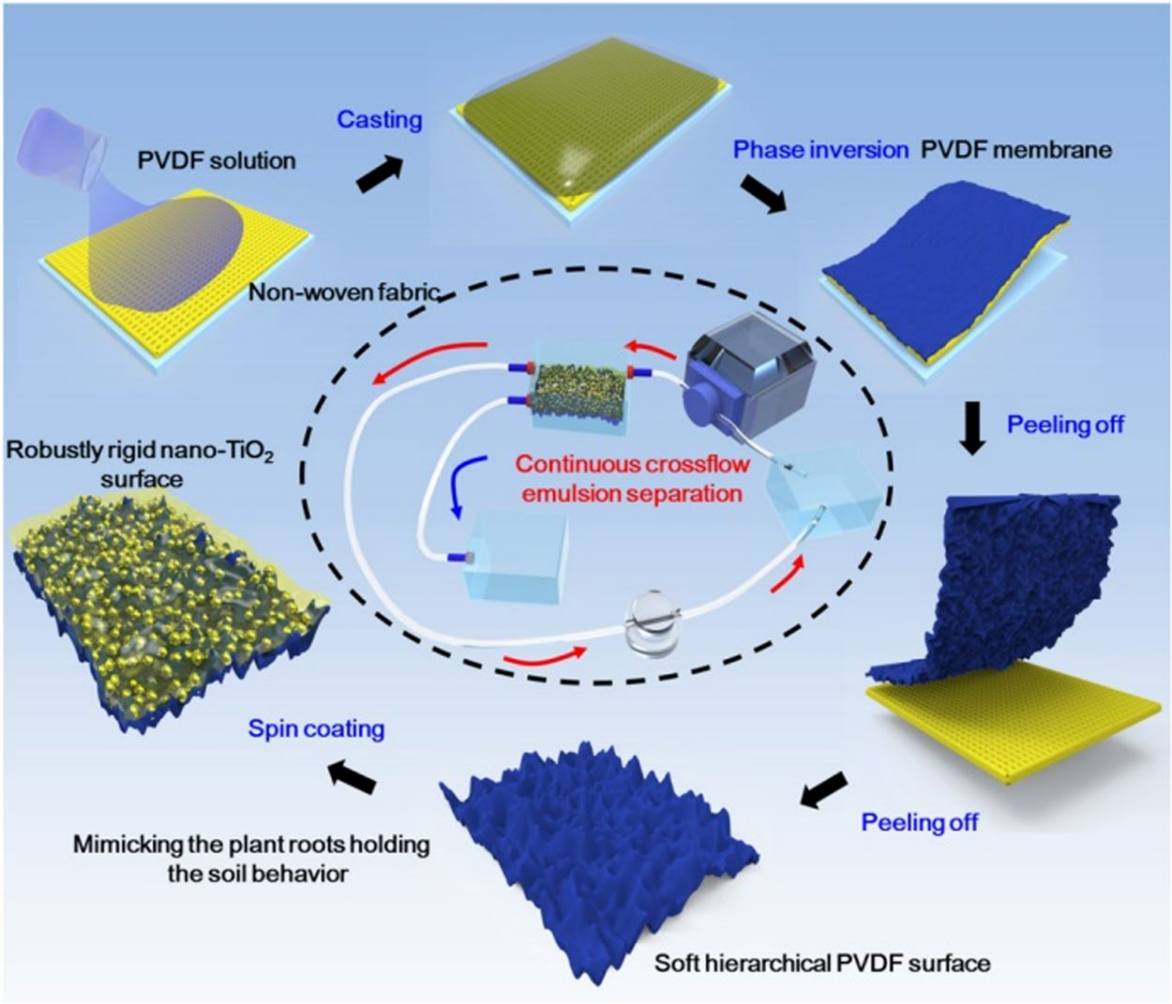
Schematic illustration of robust superhydrophobic surface based on PVDF membrane via the caging effect of confined micro-/nano- structure.

Herein, we mimicked the pant root holding the soil behaviour to immobilize the superwetting nanoparticles to develop a flexible PVDF membrane with robust superwetting surface capable of separating stabilized oil/water emulsions in a continuous crossflow mode. The production of the membrane is schemed in Fig. 1: (1) Construction of soft micro-/nano- hierarchical surface as the root-like model based on model PVDF membrane via phase inversion and peeling off from non-woven fabric, (2) Synthesis of Perfluorosilane-modified titanium dioxide (TiO_2_) nanoparticles. Details about synthetic chemistry, structural characteristic and superhydrophobicity of the perfluorosilane-modified TiO_2_ nanoparticles are summarized in Supporting Information (Figure S1), (3) Seeding of superhydrophobic TiO_2_ nanoparticles on PVDF membrane surface via the caging effect of root-like micro-/nano- pores to produce the robust superhydrophobic surface for various water-in-oil emulsions separation. The root-immobilized superhydrophobic nano-TiO_2_ coating endowed the flexible PVDF membrane with excellent superhydrophobicity and superoleophilicity as well as robustly ultralow water adhesion.

Meanwhile, we estimated the robustness of superhydrophobicity for the nano-TiO_2_ root-immobilized PVDF membrane after the vigorous sandpaper abrasion, long term hydraulic pressure, aggressive chemical corrosion, high (~180 °C) or low temperature (liquid nitrogen) torture and surfactant contamination. Moreover, we also prepared the superhydrophilic nano-TiO_2_ to decorate the root-like PVDF membrane surface, and demonstrated the superhydrophilic robustness of the as-prepared PVDF membrane in detailed under the harsh crossflow water damage. Finally, the crossflow filtration as a continuous separation model was used to confirm the separation efficiency of water-in-oil emulsion and oil-in-water emulsion for the superhydrophobic and superhydrophilic nano-TiO_2_ root-immobilized PVDF membrane, respectively.

## Results and Discussion

### Morphology, chemical component and 3D structure of the rigid membrane

**Figure 2:**
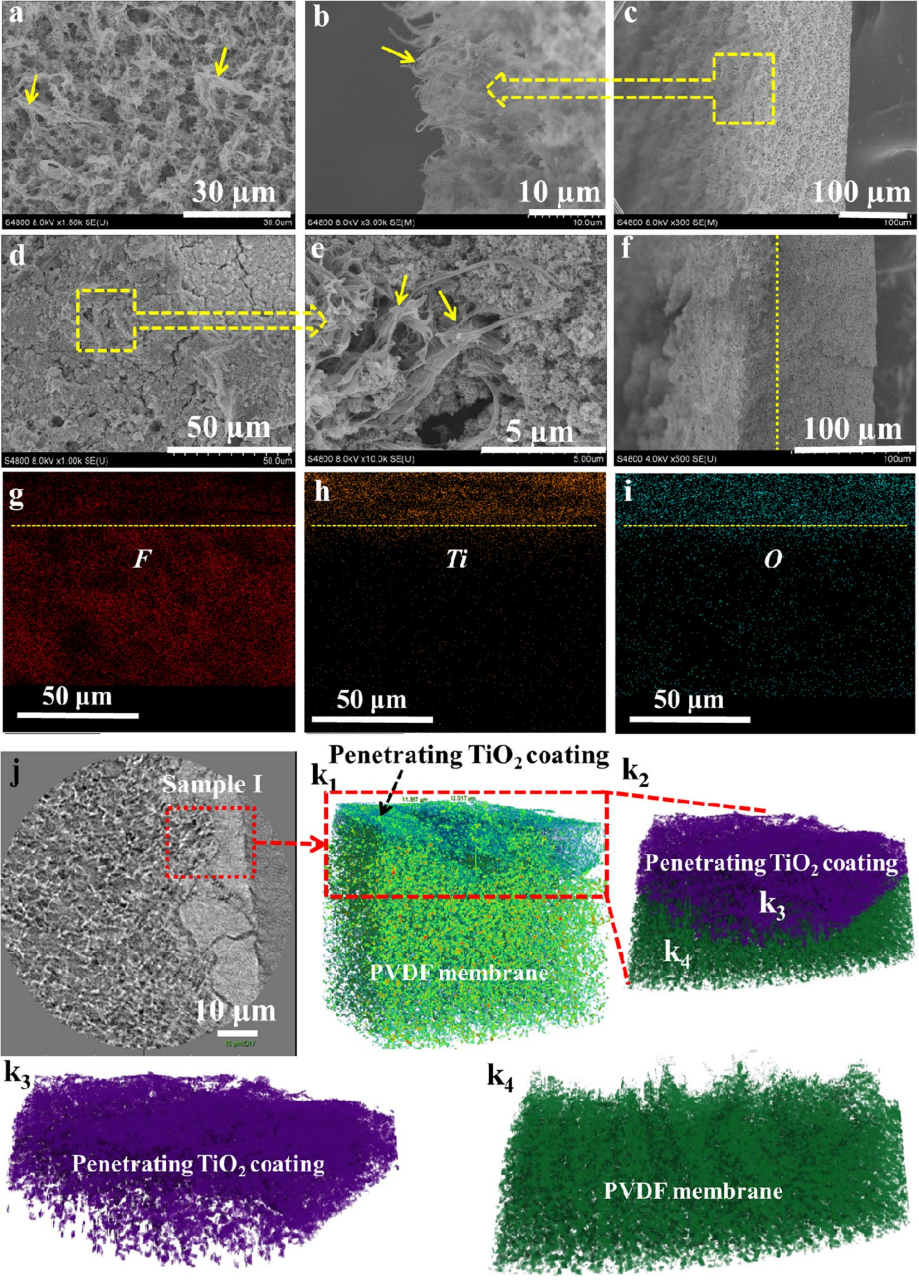
Structure and chemical components of the pristine PVDF membrane (**a**–**c**) and nano-TiO_2_ manipulated membrane (**d**–**f**). (**a**,**c**) SEM images of the pristine PVDF membrane surface and cross-section. (**b**) Enlarged SEM image of the pristine PVDF membrane cross-section. (**d**,**f**) SEM images of the nano-TiO_2_ manipulated membrane surface and cross-section. (**e**) Enlarged SEM image of the nano-TiO_2_ manipulated membrane surface. (**g**,**h** and **f**) EDX mapping of F, Ti and O elements distribution for nano-TiO_2_ manipulated membrane cross-section. (**j**) Overview of nano-TiO_2_ manipulated membrane for nano-CT. (k_1_–k_4_) 3D reconstructed and stripped microstructure of Sample I.

To achieve the robustness of the superhydrophobicity, understanding the interfacial combination between flexible PVDF membrane and rigid TiO_2_ nanoparticles, is important. Figure 2a–c shows the SEM images of pristine PVDF membrane surface full of confined microspores. From the amplification of the cross section in Fig. 2b, large quantities of cilia-like micro/nano-fibrils, which could be described as the plant roots, stretch out of the PVDF membrane surface with length arranged from 10 to 20 μm. The templating of NWF plays a distinct role in the formation of hierarchically root-like topography of PVDF membrane^25^. Meanwhile, the synergism of raspy peaks and confined microspores provide a high roughness ~12.4 μm to obtain superhydrophobicity with the water contact angle about ~153° for the pristine PVDF membrane surface (Figure S2). In accordance with previous studies, superhydrophobic surface can be directly produced by enhancing the surface roughness based on low surface energy materials^26–28^.

Interestingly, the flexible hierarchical surface can function like the plant roots holding the soil behavior to capture, confine and cage perfluorosilane TiO_2_ nanoparticles to form the rigid superhydrophobic coating as shown in Fig. 2d,e. This unique behavior of our prepared PVDF membrane was not found in the other literatures. The newly assembled perfluorosilane TiO_2_ coating with the roughness of 7.6 μm (Figure S2) interpenetrated with PVDF membrane (Fig. 2f), exhibited the superhydrophobicity with the contact angle of ~154° (Figure S2). In contrast with the perfluorosilane TiO_2,_ the pristine TiO_2_ could also form a layer on the PVDF membrane surface with the roughness of 7.8 μm. However, the corresponding surface exhibited the hydrophilicity with the contact angle of ~36° as demonstrated in Figure S2. As revealed by the element mapping in Fig. 2g–i, the perfluorosilane TiO_2_ nanoparticles are uniformly distributed on the pristine PVDF membrane surface and form a compacted nano-layer with the thickness of ~20 μm, which contributed to the superhydrophobicity of PVDF membrane. Furthermore, the inorganic-organic phase interface was explored by three dimension nano-CT in Fig. 2j,k_1_–k_4_. It was found that TiO_2_ coating is not simply covered on PVDF membrane surface, but perfectly penetrated into the confined microspores and cilia-like micro/nano-fibrils to form an organic-inorganic hybridization interface. The interface was reconstructed and stripped into the organic substrate and inorganic coating in a three dimensional view as shown in Fig. 2k_3_,k_4_ and Movie S1. The chemical variation of the pristine PVDF membrane and the rigid PVDF membrane decorated by perfluorosilane TiO_2_ nanoparticles were analyzed by XPS and FTIR as shown in Figure S3 and Table S1. It indicated that the existence of FOTS on the TiO_2_ coating endowed the PVDF membrane surface with superhydrophobicity.

### Robust superhydrophobicity determination of rigid PVDF membrane surface

**Figure 3:**
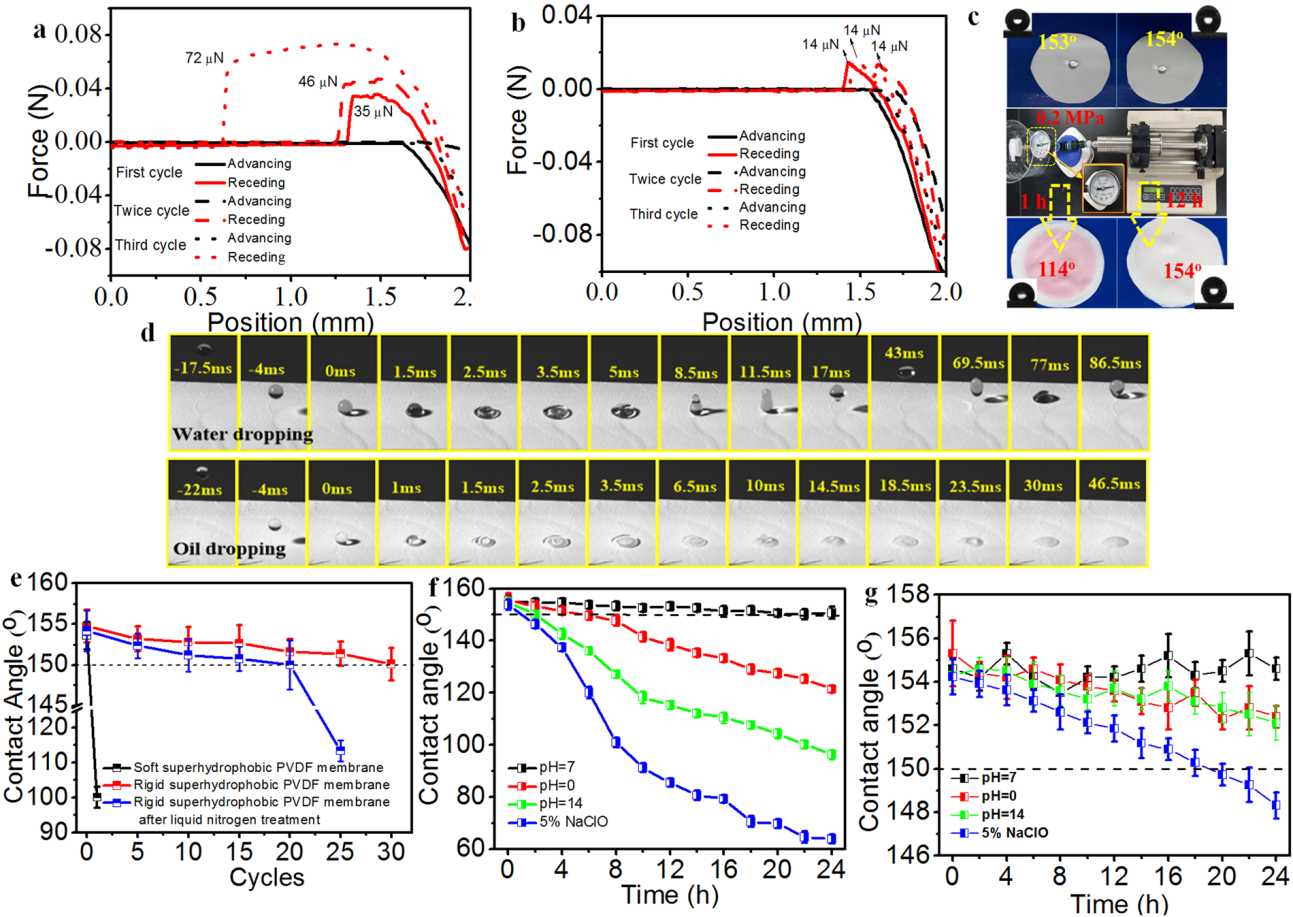
(**a**) The adhesion force of the soft PVDF membrane measured with three cycles. (**b**) The adhesion force of the rigid PVDF membrane measured with three cycles. (**c**) The hydraulic pressure resistance tests of the soft superhydrophobic PVDF membrane (left) and rigid superhydrophobic PVDF membrane (right) at 0.2 MPa. (**d**) High-speed camera photographs of water and oil (toluene) dropping on the rigid PVDF membrane, respectively. (**e**) Water contact angles (WCAs) versus sandpaper abrasion cycles for the soft and rigid superhydrophobic PVDF membrane surfaces with and without liquid nitrogen treatment, respectively. (**f**,**g**) WCAs versus immersion time in strong acid, strong alkali and strong oxidant NaClO aqueous solution for the soft and rigid superhydrophobic PVDF membrane surfaces, respectively.

To better understand the robustness of rigid PVDF membrane surface, adhesion forces of both soft PVDF and rigid PVDF membrane surface during water droplet contact procedure were dynamically measured with three cycles in the same site. As shown in Fig. 3a, when squeezing an water droplet (3 μL) against membrane surface and lifting it up in air subsequently for three cycles, there was no obvious deformation of the water droplet observed in case of rigid superhydrophobic PVDF membrane surface. The adhesive force during the lifting process of three cycle tests is almost unchanged with a very low value of 14 μN, indicating a stable and excellent water-repellent property. In contrast, an obvious deformation is observed in case of soft superhydrophobic PVDF membrane surface during three cycles’ tests as shown in Fig. 3b, and the adhesive force increased to 72 μN for the third cycle. This difference was mainly caused by the creeping deformation of the hierarchical micro-/nano- structure. The delicate polymeric membrane surface was easily crashed even by slight pressing, which resulted in the decay of water repellency.

High hydraulic pressure (0.2 MPa) was further applied onto the soft and rigid PVDF membrane with superhydrophobicity for certain time, respectively. As shown in Fig. 3c, the contact angle of soft PVDF membrane declined to 114° after 1 h hydraulic pressure and the membrane was tanned pink, indicating the loss of superhydrophobicity and anti-adhesion. In comparison, the rigid PVDF membrane manipulated by nano-TiO_2_ maintained its superhydrophobicity and resisted the intrusion of water under 0.2 MPa for 12 h. And the membrane still showed the high contact angle of 154° and was not contaminated by dye. Thus, the interface strengthening strategy conquered the inherent creep deformation of polymeric surface and offered a robust superhydrophobic surface.

We further investigate the interaction between liquid droplet (water and oil) and membrane surface through the droplet bouncing test^17,29,30^. Water droplet with a volume of 5.0 μL was impinged onto the membrane surface from the height of 100 mm, and the bouncing process was recorded by the high-speed camera (Fig. 3d, Movie S2). In Fig. 3d, the water droplet hit the rigid PVDF membrane surface and bounced off immediately, indicating the excellent superhydrophobicity and the low adhesion. The drop’s potential energy was transformed into vibrational energy, allowing the droplet to rebound before it underwent damped oscillations and finally stayed on the membrane surface with a quasi-spherical state^31,32^. Contrariwise, the oil droplet contacted, adhered, expanded and c the membrane surface instantaneously (Fig. 3d and Movie S3), indicating the superoleophilicity. These results revealed that the rigid PVDF membrane surface exhibits stable superhydrophobicity under hydraulic pressure due to the assembly of perfluorosilane TiO_2_ coating.

The traditional polymeric membrane with micro-/nano- structure surface was vulnerably damaged by the unexpected abrasion^14,15,17^, which resulted in the malfunction of superhydrophobicity, such as polytetra-fluoroethylene (PTFE)^1,33^, polyvinylidene fluoride (PVDF)^10–12^, and polystyrene (PS)^9^. We comprehensively evaluated the superhydrophobic robustness under extremely strict damage test including finger-wipe (Movies S4), sandpaper abrasion even after liquid nitrogen quenching (Fig. 3e, Movies S5 and S6), high temperature (180 °C) and pressure treatment (Figure S4, Movie S7) and chemical corrosion of strong acid (pH = 0), strong alkali (pH = 14) and strong oxidant sodium hypochlorite (5% NaClO) (Fig. 3e,f) for both soft and rigid super-hydrophobic PVDF membrane, respectively. As shown in Movie S4, the soft PVDF membrane surface easily lost its superhydrophobicity as the water droplets tightly adhered and formed an obvious stain on the surface, demonstrating the vulnerability of the polymeric rough surface. However, the PVDF membranes manipulated by perfluorosilane TiO_2_ coating showed robust superhydrophocity resistant to finger-wipe. The sandpaper abrasion was carried out to further determine the robustness of the rigid superhydrohobic surface. One abrasion cycle is shown in Movie S5, which guarantees the sufficient abrasion longitudinally and transversely. As shown in Fig. 3e, the WCA still keeps above 150° even in 30 abrasion cycles for the rigid superhydrophobic PVDF membrane surface. This result was further proved by Movie S6 after quick abrasion with the ultralow temperature treated rigid PVDF membrane for 50 times. It displayed that the rigid perfluorosilane TiO_2_ interface was not comprised by frequent abrasion. In comparison, the WCAs of soft superhydrophobic PVDF membrane were dramatically decreased to 100° after only one abrasion cycle due to the inherent fragility of polymeric micro-/nano-structures, as revealed in Fig. 3e. In this work, it is worthy being noted that no extra binders such as double side tapes^17^, dopamine^34^ and reactive coupling agents^35,36^ were involved to chemically or physically bind the interface. The caging and confining effect of the fibrils and microspores was thought to restrain the size-matching nanoparticles firmly mimicking the plant roots holding soil behaviour^16,17^. The reconstructed nano-CT in Fig. 2 (k_3_,k_4_) clearly showed the perfect interface combination between the raspy texture and nanoparticles. To further explore the limit of robust superhydrophobic interface, we frozen our membrane in liquid nitrogen for 24 h and then rubbed the membrane with the sandpaper. Within 20 abrasion cycles, the rigid PVDF membrane with perfluorosilane TiO_2_ interface still exhibited superhydrophobicity with the WCA around 150° in Fig. 3e. Afterwards, the WCA was gradually decreased and eventually dropped below 110° in 25 abrasion cycles. The extreme freezing far below the glass transition temperature of PVDF (−39 °C) caused the brittleness of the fibrils and weakened the bonding of nanoparticles accordingly. Nevertheless, the robust resistance of the perfluorosilane TiO_2_ interface on PVDF membrane to this extreme abrasion still impressed us surprisingly.

Besides the physical destructive tests, the interface chemical stability of the soft and rigid PVDF membrane was thoroughly examined by the corrosion of strong acid, strong alkali and strong oxidant sodium hypochlorite (NaClO) for long term, respectively. Considering of that, the membranes were immersed in an aqueous HCl solution (pH = 0), an aqueous NaOH solution (pH = 14), and an aqueous NaClO solution (5 wt%) for 24 h, respectively. As shown in Fig. 3f, the soft PVDF membrane lost its superhydrophobicity with WCA decreasing below ~150° after the strong acid treatment for 8 h, strong alkali treatment for 4 h and NaClO treatment for only 2 h. By contrast, in Fig. 3g, the rigid PVDF membrane perfluorosilane TiO_2_ interface is resistant to the harsh corrosion of acid, alkali and NaClO. The WCA almost keeps above ~150 °C during the 24 h immersing process, showing highly durable superhydrophobicity. There have long been investigated that NaOH and NaClO could be detrimental to PVDF chemical structure. In contrast with NaOH, the usage of NaClO causes a more detrimental effect towards the stability of PVDF membrane. Therefore, as shown in Fig. 3f,g, the robustness of superhydrophobicity is weakest in the aqueous NaClO solution for the as-prepared PVDF membranes. However, even though the rigid PVDF membrane treated in NaClO, it exhibited a much longer superhydrophobic time than that of soft PVDF membrane. Thus, we can conclude that the fragile micro-/nano- structure of polymeric surface was seriously deteriorated by the chemical attack. But, the superhydrophobicity and the micro-/nano- structure could be well protected by the robust nano-TiO_2_ interface in the rigid PVDF membrane.

It is clear that the fragile micro-/nano- structure of polymeric surface was seriously deteriorated by the physical and chemical attack. But, the superhydrophobicity could be perfectly protected by the robust TiO_2_ coating in the rigid PVDF membrane via the caging and confining effect of fibrils and microspores mimicking the plant roots holding the soil behaviour, which is promising for the continuous separation of water-in-oil emulsions in the harshly chemical environment.

### Continuous water-in-oil emulsion separation with the rigid superhydrophobic PVDF membrane

**Figure 4:**
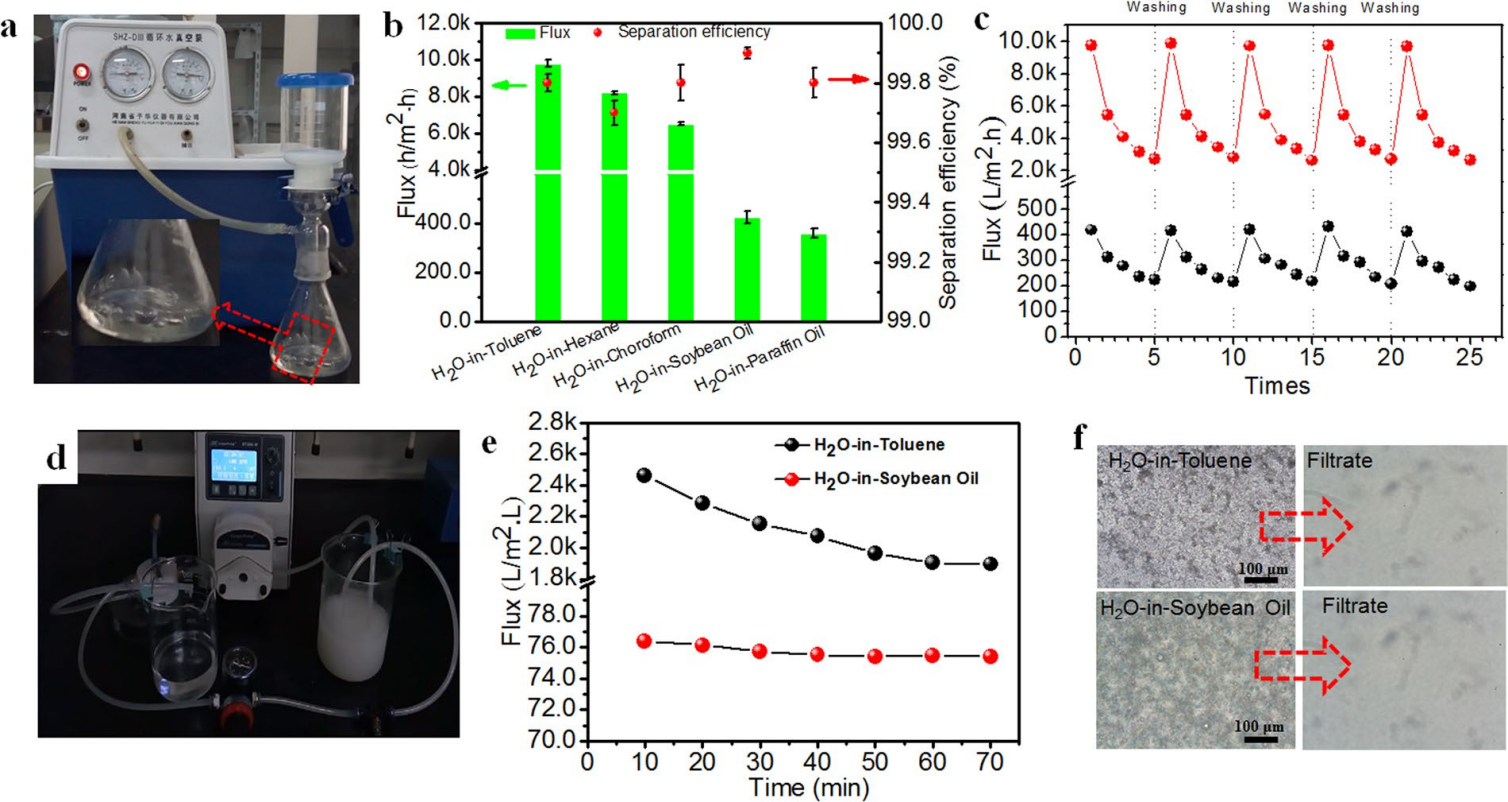
(**a**) Dead-end separation device. (**b**) Flux and separation efficiency of the water-in-oil emulsions with the dead-end flow mode. (**c**) Fouling and recovery of the rigid membrane for H_2_O-in-Toluene (black symbol) and H_2_O-in-Soybean Oil (red symbol) emulsion with batch operation. (**d**) Continuous crossflow filtration separation device. (**e**) Flux of H_2_O-in-Toluene (red symbol) and H_2_O-in-Soybean Oil (black symbol) emulsions with time. (**f**) Filtrate observation for H_2_O-in-Toluene and H_2_O-in-Soybean Oil emulsions after crossflow filtration.

In separation experiment, five stable H_2_O-in-oil emulsions including span80-stabilized H_2_O in toluene, chloroform, hexane, soybean oil, and paraffin oil emulsion was prepared. The droplet size distribution and viscosity of five emulsions were shown in Table S2 and Figure S5. Herein, water-in-toluene emulsion was chosen as a sample for the dead-end flowing test. After separation, the milky emulsion turned to the transparent toluene (Fig. 4a and Movie S8). Therefore, the rigid superhydrophobic PVDF membrane can effectively separate stabilized water-in-oil emulsion via batch filtration. From Fig. 4b, it was observed that the as-prepared PVDF membrane presented high flux of 9800 ± 20 L·m^−2^·h^−1^, 8200 ± 72 L**·**m^−2^ **·**h^−1^, and 6500 ± 85 L**·**m^−2^ **·**h^−1^ for water-in-toluene, water-in-hexane, and water-in-chloroform emulsion, respectively. For water-in-soybean oil and water-in-paraffin oil emulsions, the membrane flux is 426 ± 23 L**·**m^−2^ **·**h^−1^ and 360 ± 19 L**·**m^−2^ **·**h^−1^, respectively, which was much lower than the above three emulsions due to higher viscosity^9^. The purity of filtrates was characterized by optical microscope images and a coulometer, as shown in Figure S5. All filtrates were totally clear and transparent in contrast to the milky emulsions. The purity of all filtrates, which filtrated from both light and viscous water-in-oil emulsions, were all above 99.5% (as shown in Fig. 4b), demonstrating the high separation efficiency of our membrane. Moreover, the flux of both low viscous water-in-toluene and high viscous water-in-soybean oil emulsions decreased with time because the demulsified water accumulated and formed a water barrier layer to hamper oil flow in the dead-end filtration. However, the flux can be almost recovered after alcohol washing after multiple operations as shown in Fig. 4c.

A crossflow filtration was implemented to assess the continuous separation of water-in-oil emulsion as shown in Fig. 4d and Movie S9. The rigid superhydrophobic PVDF membrane showed stable permeability for both water-in-toluene and water-in-soybean oil emulsions as depicted in Fig. 4e. In continuous 70 minutes’ operation without any residence or wash, the membrane showed a flux of 1900 L·m^−2^·h^−1^ for water-in-toluene and 75 L·m^−2^·h^−1^ for water-in-soybean oil respectively. A high purity of permeate was obtained as shown in Fig. 4f. The stable permeability of stabilized emulsions demonstrated the excellent antifouling property, robust superhydrophobicity of rigid PVDF membrane manipulated by nano-TiO_2_. Comparing to the previous studies concerning water-in-oil emulsion separation^1,9,10,13^, it was the first success of continuous and highly efficient separation of both light and viscous surfactant stabilized water-in-oil emulsions in practical by robust superhydrophobic PVDF membrane. By contrast, the soft superhydrophobic PVDF membrane (Movie S9, **left**), which unstrengthen by the rigid nanoparticles, lost its efficiency on the separation of water-in-oil emulsion immediately while launching the cross-flow system. Thus, we can believe that the coral tentacles-immobilized the hydrophilic nano-TiO_2_ layer plays a crucial role on the continuous separation of water-in-oil emulsion for the polymer membranes. In addition, the robust superhydrophilic PVDF membrane manipulated by hydrophilic nano-TiO_2_ was also prepared through the similar interface strengthen strategy. The as-prepared PVDF membrane can be applied in toluene-in-water emulsion separation with high permeate flux (~1700 L·m^2^·h) and separation efficiency (96%) in the continuous crossflow separation mode as shown in Figure S6 and Movie S10.

### Uniqueness of the rigid PVDF membrane for the continuous emulsion separation

**Figure 5:**
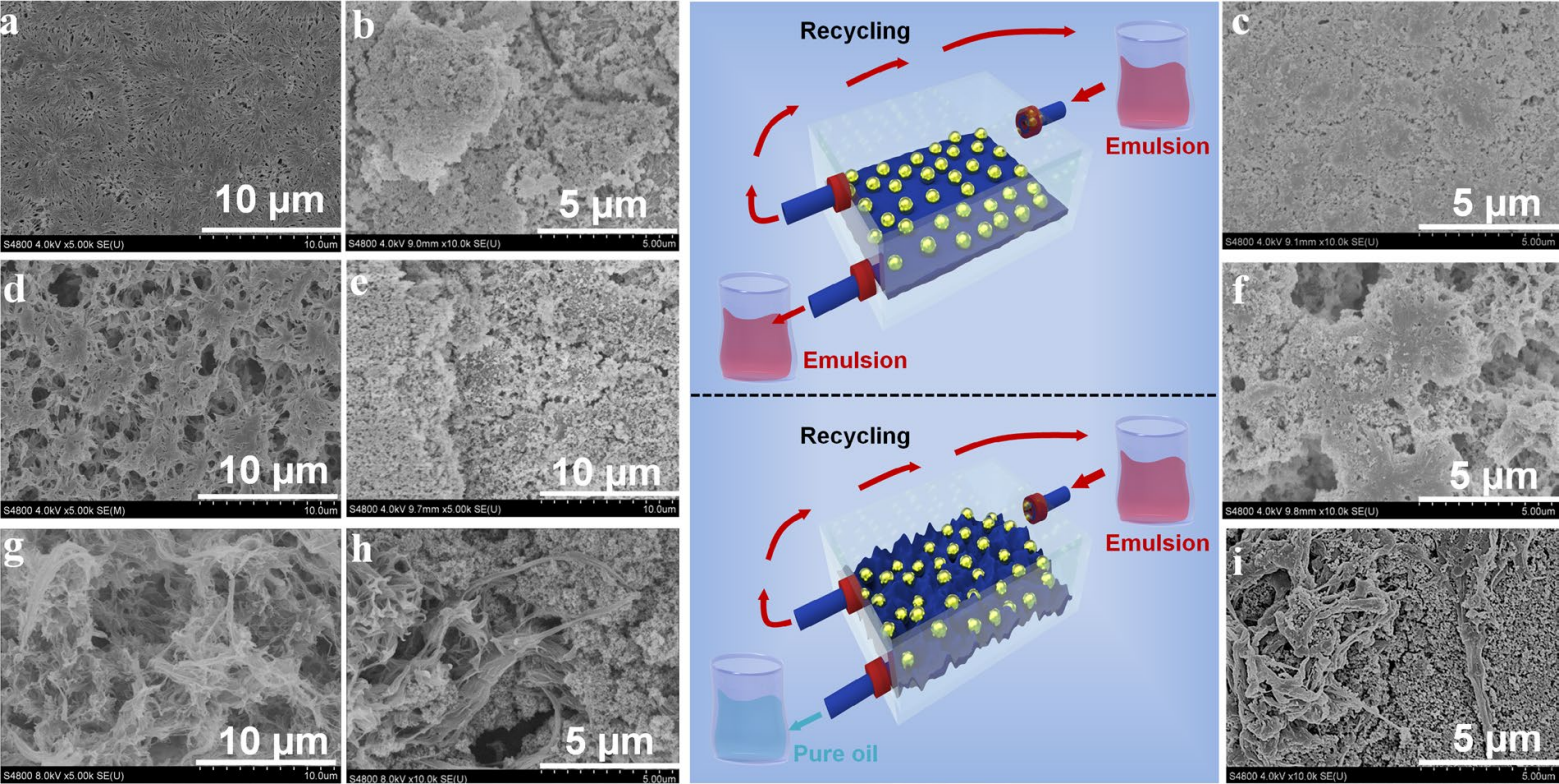
The surface SEM images of (**a**) PVDF-1 membrane, (**b**) PVDF-1 membrane coated with perfluorosilane nano-TiO_2_, (**c**) TiO_2_-coated PVDF-1 membrane after 5 min continuous flow through of water-in-toluene emulsion, (**d**) PVDF-2 membrane, (**e**) PVDF-2 membrane coated with perfluorosilane nano-TiO_2_, (**f**) TiO_2_-coated PVDF-2 membrane after 5 min continuous flow through of water-in-toluene emulsion, (**g**) Raspy PVDF membrane, (**h**) Raspy PVDF membrane coated with perfluorosilane nano-TiO_2_, and (**i**) Robust TiO_2_-inlayed PVDF membrane after 70 min continuous flow through of water-in-toluene emulsion.

In order to reveal the unique function of plant roots-like fibrils on raspy surface in immobilizing the size-matched nanoparticles, the smooth PVDF-1 (pore size ~0.17 µm) and PVDF-2 (pore size ~0.73 µm) membrane (Fig. 5a,d) were both utilized to load the perfluorosilane nano-TiO_2_ coating. However, the loosely attached coating from PVDF-1 and PVDF-2 almost disappeared due to the shearing of cross flow emulsion, as exhibited in Fig. 5c,f). Correspondingly, both PVDF-1 and PVDF-2 membranes decorated by perfluorosilane nano-TiO_2_ could not realize the continuous water-in-oil emulsion separation even for few seconds as shown in Movie S11. However, even after continuous crossflow of water-in-toluene emulsion after 70 min, the perfluorosilane nano-TiO_2_ coating was still firmly retained on the raspy PVDF membrane surface due to the caging and confining effect of numerous fibrils and microspores as exhibited in Fig. 5g–i. It demonstrated the advance of the hierarchical micro-/nano- texture including stretching fibrils and size-matching pores of polymeric membranes in immobilizing the rigid inorganic nanoparticles to construct a strengthen interface for continuous oil/water emulsions separation.

### Morphology of the rigid PVDF membrane with the root-immobilized superhydrophilic nano-TiO_2_

**Figure 6:**
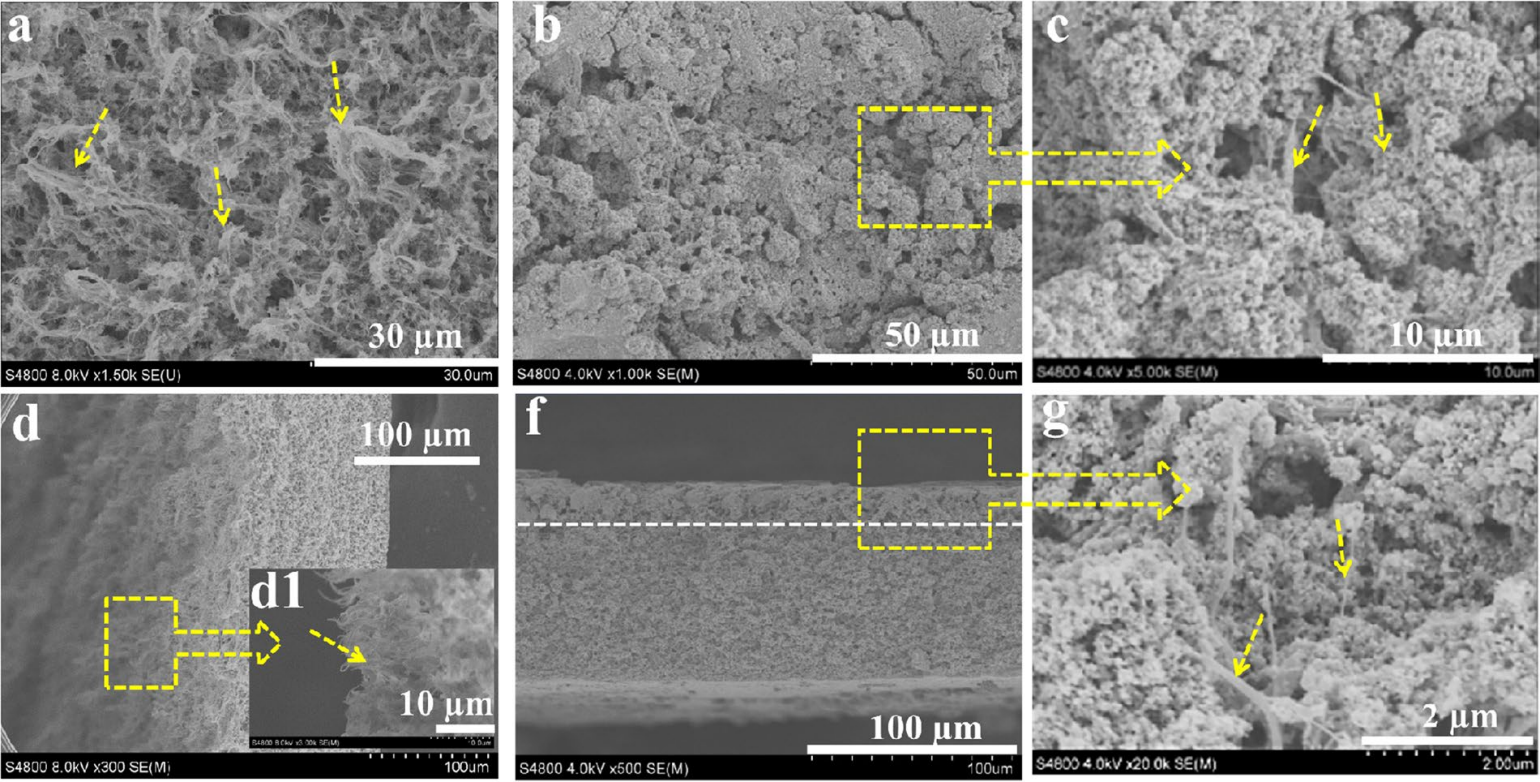
(**a**,**d**) SEM images of the pristine membrane surface and cross-section. (d1) Enlarged SEM image from the top zone of the pristine membrane cross-section. (**b**,**f**) SEM images of the hydrophilic nano-TiO_2_ coated membrane surface and cross-section. (**c**,**f**) Enlarged SEM image from the hydrophilic nano-TiO_2_ coated membrane surface.

By means of the plant root holding the soil mold, we also prepared the superhydrophilic PVDF membrane. Figure 6 shows the SEM images of the pristine PVDF membrane hierarchical surfaces and cross-sections after NWF peeling off. The pristine PVDF membrane surface is rather raspy with microspores [Fig. 6a] and stretching nanofibrils [Fig. 6d,d1]. The size matched superhydrophilic TiO_2_ nanoparticles were captured by the stretching nanofibrils behaving like plant roots and confined by the microspores to form the robust superhydrophilic coating with the thickness of ~20 µm on the pristine PVDF membrane, which can be identified in Fig. 6b,f. The plentiful micro-/nano- structure offered a high surface area to accommodate nanoparticles. Especially from the higher magnification image in Fig. 6c,g, it is clearly found that the hydrophilic TiO_2_ nanoparticles was immobilized by the porous root-like surface and formed the rigid inorganic coating.

### Superwettability, permeation flux, separation efficiency and robustness of the rigid superhydrophilic PVDF membrane

**Figure 7:**
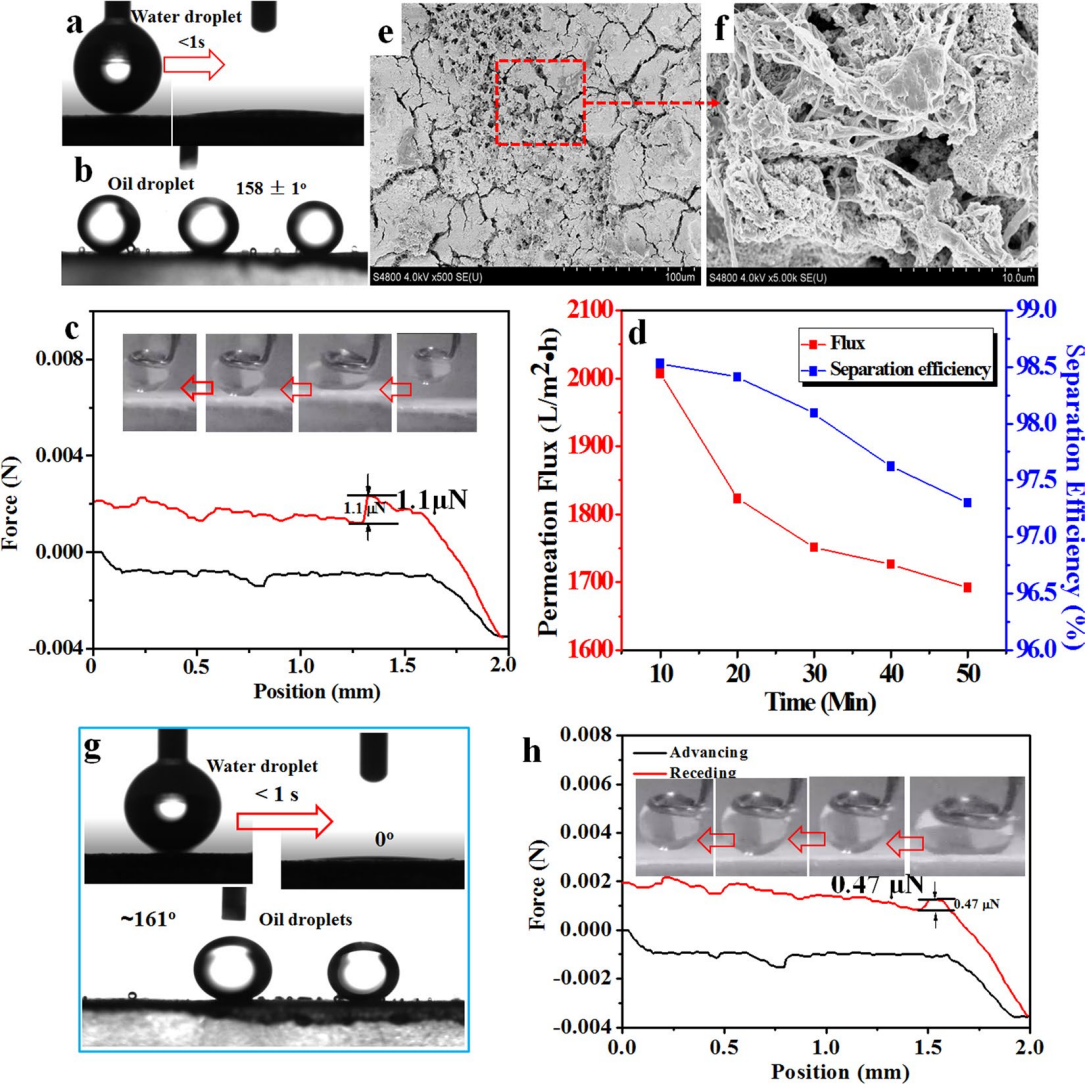
(**a**,**b**) The water contact angle and underwater oil droplet contact angle of the rigid superhydrophilic PVDF membrane manipulated by hydrophilic nano-TiO_2_, respectively. (**c**) Real-time recorded force-distance curves for rigid superhydrophilic PVDF membrane during the dynamic adhesion measurements. (**d**) The permeation flux and separation efficiency of the as-prepared rigid superhydrophilic PVDF membrane. (**e**,**f**) SEM images of the rigid superhydrophilic PVDF membrane after 2 h continuous crossflow separation. (**g**) The water contact angle and underwater oil droplet contact angle of the rigid superhydrophilic PVDF membrane after 2 h continuous crossflow separation, respectively. (**h**) Real-time recorded force-distance curves for the washed rigid superhydrophilic PVDF membrane surface during the dynamic adhesion measurement.

As shown in Fig. 7a, it is very interesting that the soft superhydrophobic PVDF membrane surface was converted into superhydrophilicity with the water contact angle instantaneously dropping to zero in 1 s after the immobilization of superhydrophilic nano-TiO_2_. Meanwhile, the superhydrophilic PVDF surface displayed a high underwater oil contact angle (OCA) of 158 ± 1°, and no obvious deformation of the oil droplet with the very ultralow adhesion force of 1.1 μN was observed during the contacting test [Fig. 7b,c], showing the outstanding superoleophobicity underwater. This is because that the water trapped in the hierarchical TiO_2_ coating formed a stable hydration layer to reduce the contacting opportunity between oil and PVDF membrane, which therefore provided a robust membrane for oil-in-water emulsion separation.

The permeability and separation efficiency of oil-in-water emulsion were exhibited in Fig. 7d and Movie S10. It was observed that the rigid superhydrophilic PVDF membrane maintained a flux over ~1700 L·m^−2^·h^−1^ and separation efficiency over 96% for toluene-in-water emulsion, respectively. The Gas Chromatograph Mass Spectrometer (GC-MS, Aglient, 7890B-5977A, America) was taken to characterize the separation efficiency of oil-in-water emulsions^37^. Thus, relying on the robust superwettability of nano-TiO_2_ coating, the rigid superhydrophilic PVDF membrane can successfully realize the continuous separation of water-in-oil emulsion. Moreover, we also tested the robustness of the rigid superhydrophilic PVDF membrane surface via the long-term crossflow separation. As shown in Fig. 7e,f, an apparent superhydrophilic TiO_2_ coating, which anchored by the root-like PVDF structure, was still preserved on the PVDF membrane surface even after 50 min continuous crossflow washing. Meanwhile, as exhibited in Fig. 7g, when the water droplet and oil droplet contacted the washed rigid superhydrophilic PVDF membrane, the water contact angle was immediately dropped to 0° less than 1 s and the oil contact angle under water is ~161°, respectively, indicating the superhydrophilicity and underwater superoleophobicity. Furthermore, as demonstrated by the dynamic adhesion test in Fig. 7h, the washed rigid superhydrophilic PVDF membrane also exhibited the ultra-low oil adhesion force (0.47 μN) under water. It was thought that the polymeric micro-/nano- texture played a crucial role of anchoring the hydrophilic nanoparticles to maintain the robust superhydrophilicity of polymer membrane.

## Conclusion

In summary, we have developed a robust superhydrophobic and superhydrophilic surface based on PVDF membrane. Rigid perfluorosilane nano-TiO_2_ was perfectly inlayed into the micro-/nano- texure via the confining and caging effect mimicking the plant roots holding the soil behavior^38,39^. To the best of our knowledge, the nanoscaled interfacial combination of rigid nanoparticles and flexible membranes was first revealed by our works. The robust superhydrophobic surface could tolerate the extremely physical damage e.g. hydraulic pressure, finger-wipe, sandpaper abrasion after liquid nitrogen quenching, high temperature torture, and harsh chemical corrosion including strong acid, alkali, and NaClO. The surface robustness endowed the membrane with continuous separation for surfactant stabilized water-in-oil emulsions. PVDF membrane with robust superhydrophilic surface was also fabricated via the root-immobilization of superhydrophilic nano-TiO_2_. The as-prepared superhydrophilic PVDF membrane showed stable superhydrophilicity, underwater superoleophobicity and ultra-low oil droplets adhesion force. With the robust superhydrophilic function, PVDF membrane successfully achieved the high efficient separation in stabilized oil-in-water emulsion via the continuous crossflow model. The versatile strategy overcomes the instability of traditional polymeric micro-/nano- interfaces and can function on various polymeric membranes to achieve robust interfaces for oil/water separation, interface catalysis, interface exchange, interface conduction etc.

## Experimental Section

### Materials

PVDF (Kynar 761-A, Arkema) was purchased from Arkema Company (Changshu, China) and dried at 80 °C for 24 h before use. Triethylphosphate (TEP), absolute alcohol, chloroform, hexane, paraffin oil, toluene, sodium hydroxide and hydrochloric acid were purchased from Sinopharm Chemical Reagent Co. Ltd., China. Span 80, soybean oil, methylene blue, and congo red were purchased from Aladdin Reagent Co., Ltd., China. PET non-woven fabric (90 g**·**m^−2^) was supplied by Jitian Co., Japan. 1 H, 1 H, 2 H, 2H-perfluorooctyltriethoxysilane (FOTS) was purchased from Wuhan Silworld Chemical Co., Ltd (China). Titanium Oxide (P25) was supplied by Degussa (Germany). N-vinyl-2-pyrrolidone (NVP) (AR), Vinyltriethoxysiliane (VTES) (AR), and 2, 2′-azobis (2-methylpropionitrile) (AIBN) (AR) were bought from Aladdin (China).

### Fabrication of the soft hierarchical PVDF membrane

15 g PVDF (Kynar 761-A, Arkema) is dissolved in 85 g triethyl phosphate (TEP) at 80 °C for 24 h under 300 rmp mechanical stirring. After that, the solution was deaerated under reduced pressure and then heated to 80 °C to obtain uniform PVDF casting solution. Accomplished by that, the above PVDF solution was spread onto the special PET non-woven fabric (NWF) (90 g·m^−2^) by a casting knife with the thickness of 200 μm. The nascent membrane was immediately immersed into coagulation bath composed of TEP/water mixture (v/v: 5/5) for 5 s, and then moved to pure water bath at room temperature. After total solidification, the membrane was transferred to the fresh deionized water for 24, and dried in the air to obtain the NWF supported PVDF membrane. Lastly, the PVDF membrane with the mult-scale surface structure was fabricated via the NWF peeling off and marked as the pristine PVDF, as schemed in Fig. 1. As a contrast, the PVDF membrane was also prepared without the NWF supporting via the NIPS according to the above method, in which, its top surface and bottom surface were marked as PVDF-1 and PVDF-2, respectively.

### Fabrication of the rigid superhydrophilic PVDF membrane and its separation in the Oil-in-Water emulsion

The superhydrophobic TiO_2_ nanoparticles was prepared as following based on Lu’ studying:^17^ 0.6 g of 1 H, 1 H, 2 H, 2H-perfluorooctyltriethoxysilane and 2 mL deionized water were put into 100 mL of absolute ethanol, and the solution was mechanically stirred for 2 hours under 150 rmp. After that, 3 g of 25 nm fumed titanium oxide (TiO_2_) nanoparticles (Degussa, P25) was added to make a paint-like suspension after 2 h of ultrasonic treatment. Subsequently, these superhydrophobic TiO_2_ nanoparticles were immobilized onto the pristine PVDF membrane to fabricate the rigid PVDF membrane via the method of spin-coating. Lastly, the rigid PVDF membrane was dried in air for at least 12 h before testing. The preparation process of the robust PVDF membrane was schemed in Fig. 1. In contrast, the surfaces of PVDF-1 and PVDF-2 were both used to load these superhydrophobic TiO_2_ nanoparticles via spin coating.

Five types of surfactant stabilized water-in-oil emulsions including water in toluene, chloroform, hexane, soybean oil, and paraffin have been prepared. For each type of emulsion, water and oil was mixed in 1/114 (v/v) with the adding of 0.5 g of span 80 (HLB = 4.3, an emulsifer of the water-in-oil type). All stable emulsions were obtained after 24 h mechanical stirring at 250 rmp. The efficiency of the rigid PVDF membrane on water-in-oil emulsions seperation was firstly performed with a dead-end filtration system at 0.1 MPa (vacuum degree −0.1 MPa). Subsequently, the separation experiment was further carried out with a crossflow filtration system at 0.1 Mpa. The fluxes of the rigid PVDF membrane for water-in-oil emulsions were determined by calculating the volume of the permeate in unit time using the following equation 1:

1 $$Flux=\frac{V}{S\Delta t},$$

where V is the volume of the permeate in dead-end and crossflow filtration, S is the valid area of the membrane (12.56 cm^2^ and 6.15 cm^2^ used in dead-end filtration and crossflow filtration, respectively) and Δt is the testing time. For each test in dead-end filtration, 20 mL of emulsion was poured into the filter for separation before starting the vacuum pump, and five samples were measured to obtain the average value of the flux. For each test in crossflow filtration, the permeated purified oil was consecutively collected and weighted every 10 min at 0.1 Mpa until stable to calculate the flux.

### The Fabrication of Rigid Superhydrophilic PVDF membrane and its separation in the oil-in-water emulsion

Firstly, the hydrophilic P(VP-VTES) copolymer was synthesized as following: 3.75 g NVP, 2.75 VTES and 0.16 g AIBN was sequentially added into the 100 g alcohol, in which AIBN was used as the initiator^25,40^. The free radical polymerization was carried out at 65 °C with continuous stirring under a nitrogen for 24 h to obtain the P(VP-VTES)/alcohol solution. After that, 1.5 g nano-TiO_2_ was added to make a paint-like suspension after 2 h ultrasonic treatment. TiO_2_ nano-particles were decorated by the hydrophilic copolymer via the hydrolysis, condensation and crosslinking. Subsequently, these hydrophilic TiO_2_ nanoparticles were immobilized on the hierarchical surfaces of PVDF membrane via spin coating.

For oil-in-water emulsion separation, a stabilized toluene-in-water emulsion was used as a model for the as-prepared superhydrophilic PVDF membrane. For the toluene-in-water emulsion, 0.5 g tween 80 (HLB = 15) was added into toluene and oil mixture with the volume of 1/114 (v/v). All stable emulsions were obtained after 24 h mechanical stirring at 250 rmp. The flux of the superhydrophilic rigid PVDF membrane for toluene-in-water emulsion was determined by calculating the permeate volume per 10 min using the equation 1 via the crossflow filtration system at 0.1 MPa.

### Resistance to physical damage tests of the rigid PVDF membrane surface

(1) Finger-wiping test: the rigid PVDF membrane surface was wiped by a butyronitrile gloves wore finger, and then the changing of the superhydrophobicity was characterized by an OCA20 system (Data-physics, Germany), (2) Sandpaper abrasion test: the rigid PVDF membrane adhered on the glass slide via the double side tape was placed face-down to sandpaper (standard glass-paper, grit no. 3000) with the hierarchical surface, and then moved for 10 cm along the ruler with 200 g weight. Subsequently, the sample was rotated by 90° and then moved for 10 cm along the ruler. This process is defined as one abrasion cycle. After each cycle, the varying of the contact angle was recorded by the OCA20 system to evaluate the superhydrophobicity, (3) Sandpaper abrasion test after the ultralow temperature treatment: firstly, the rigid PVDF membrane was immersing into the liquid nitrogen for 24 h. Subsequently, the rigid PVDF membrane was taken out to undergo the sandpaper abrasion. The changing of the contact angle was recorded by the OCA20 system after every time sandpaper abrasion, (4) High temperature and pressure tests: the rigid PVDF membrane surface was treated by the flat vulcanizing machine (SZT-2, Huzhou Shuangli Automation Technology Equipment Co., LTD, China) with the temperature increasing from room temperature to 200 °C at a high pressure 10 MPa. The varying of the contact angle of the rigid PVDF membrane was recorded by OCA20 system with the temperature changing.

### Resistance to chemical damage tests of the rigid PVDF membrane surface

(1) the resistance to NaClO solution oxidization test: the rigid PVDF membrane with the dimension of 2 cm × 4 cm was immersing into 20 mL 5 wt% NaClO solution and maintained this process in the constant temperature vibrator (Changzhou Guoyu Instrument Manufacturing co., LTD) at 30 °C with a speed of 50 r/min for 24 h. During this process, the contact angle was recorded by OCA20 system per 2 h to observe the changing of the superhydrophobicity, (2) the resistance to acid and alkali solution tests: the rigid PVDF membrane with the dimension of 2 cm × 4 cm was immersing into 20 mL pH = 0 HCl and 20 mL pH = 14 NaOH solution, respectively, and maintained the two processes in the constant temperature vibrator at 30 °C with a speed of 50 r/min for 24 h. The contact angle was recorded by OCA20 system every 2 h to evaluate this changes of superhydrophobicity after the acid or alkali damage.

### Characterizations

SEM and 3D images were taken on a field emission scanning electron microscope (Hitachi S4800, Japan) and laser scanning confocal microscope (LSCM) (Zeiss LSM 700), respectively. Contact angles were measured by an OCA20 system (Data-physics, Germany) equipped with video capture at room temperature. At least five locations were tested in order to get the average value. The behavior of droplet motion from above was tested by using the high-speed camera at a rate of 2000 frames per second (FASTCAM Mini UX100, PHOTRON LIMITED, Japan). The adhesion forces were measured using a high-sensitivity microelectromechanical balance system (Data-Physics DCAT11, Germany). A water droplet (3 µL) was suspended with a metal cap and controlled to contact with membrane surface and then to leave at a constant speed of 0.05 mm/s. The force during the whole process was recorded. Optical microscopy images were taken on a BX 51TF Instec H601 (Olympus, Japan). The FTIR-ATR spectra were recorded using Thermo-Nicolet 6700 FTIR spectrometer (US). The XPS spectra was performed on a PHI-5000C ESCA System (US) with Mg Kα excitation radiation (*hν* = 1253.6 eV). The TGA was carried out with a thermo-gravimetric analysis (TGA, Mettler Toledo, Switzerland). The purity of the sperated oil was analyzed by a coulometer (KF831, Metrohm, Switzerland). DLS measurements were performed on a Zetasizer Nano ZS (Malvern, UK). A transmission X-ray microscope ZEISS XRADIA 800 Ultra (Nano-CT) was used to provide high resolution non-destructive 3D imaging.

## Electronic supplementary material

Supplemental material

Movie S1

Movie S2

Movie S3

Movie S4

Movie S5

Movie S6

Movie S7

Movie S8

Movie S9

Movie S10

Movie S11
