# Supplementary material for: Flexible PVDF membranes with exceptional robust superwetting surface for continuous separation of oil/water emulsions
Source: Sci Rep. 2017 Oct 26;7:14099. doi: 10.1038/s41598-017-14429-2 (PMC5658332; doi:10.1038/s41598-017-14429-2)
Supplement: Supplementary file 1 — Supplemental material [file 41598_2017_14429_MOESM1_ESM.pdf]

## Supporting Information

**Flexible PVDF membranes with exceptional robust superwetting surface for continuous separation of oil/water emulsions**

**Zhu Xiong†‡, Haibo Lin†‡, Fu Liu†*, Peng Xiao†, Ziyang Wu†, Tiantian Li†, Dehong Li†**

Based on the preparation route of **Figure S**1a, some characteristic peaks at 3008~2856 $cm^{-1}$, 1643 $cm^{-1}$, 1384 $cm^{-1}$, 1241 $cm^{-1}$, 1211 $cm^{-1}$, 1151 $cm^{-1}$ and 1068 $cm^{-1}$ ascribing to the -$CH_2$-, -$CH_3$-, and -CF- of FOTS was distinctly appeared in the FTIR spectra of FOTS-g-$TiO_2$ nanoparticles (**Figure S**1b), indicating some FOTS molecules grafted onto the surface of $TiO_2$ nanoparticles. TGA was carried out to calculate the FOTS grafting ratio at a heating rate of 10 $^oC$ $min^{-1}$ over a range of 80 $^oC$～700 $^oC$ under nitrogen atmosphere. As shown in **Figure S**1c, the final loss weight of the pristine $TiO_2$ and FOTS-g-$TiO_2$ nanoparticles was ~3% and ~12 wt%, respectively. Thus, we can obtain that the grafting yield of the FOTS was about 9 wt% in FOTS-g-$TiO_2$ nanoparticles. With the modification of FOTS, the water contact angle of $TiO_2$ nanoparticle coating on the glass plate could maintain at 154 $^oC$ for at least 60 s, which exhibited the excellent superhydrophobicity. But for the pristine $TiO_2$ nanoparticle, its water contact angle was quickly decreased from 105 to 31 $^oC$ in only 5 s, indicating the good hydrophilicity. Thus, we can conclude that the superhydrophobicity of the modified $TiO_2$ nanoparticles was fabricated by the grafted FOTS.

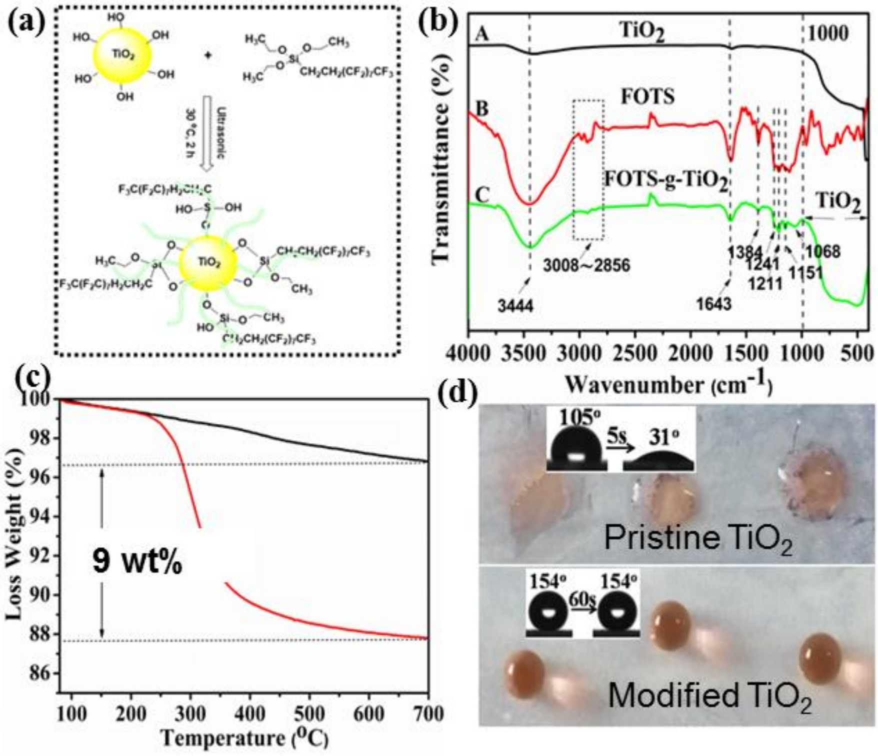


**Figure S1.** (a) The synthetic route of the superhydrophobic$TiO_2$ nanoparticles; (b) The FTIR, (c) TGA and (d) contact angle of the $TiO_2$ nanoparticles with and without modification.

There are discrete grooves of 50~100 μm in diameter disorderly distributed in the surface of the pristine PVDF membrane as shown in **Figure S2**a. From the higher magnification images, the pristine PVDF membrane surface is rather raspy with peak-to-peak roughness ~12.4 μm as shown in **Figure S2**$a_2$ and **Figure S2**c. Meanwhile, cilia-likenano-fibrils are stretching out of the surface, which could be regarded as the nano-/micro- multiscale plant roots as observed in **Figure S2**$a_1$, $a_2$. This hierarchical surface offered the pristine PVDF membrane with excellent superhydrophobicity with the water contact angle (CA) about 153$^o$ in **Figure S2**c.As shown in **Figure S2**b, $b_1$, a tight $TiO_2$ nanoparticle coating was inlayed on the surface of the pristine PVDF membrane including the discrete grooves. Whilst the image was enlarged as shown in **Figure S2**$b_2$, almost all $TiO_2$ nanoparticles are captured, confined and caged by the raspy fibrils and microspores of the pristine PVDF membrane. The newly assembled $TiO_2$ interface with the roughness of 7.6

μmexhibited superhydrophobicity with the contact angle of ~154$^{o}$ (**Figure S2**e). In contrast with the perfluorosilane$TiO_2$, the pristine $TiO_2$ could also form a layer on the PVDF membrane surface with the roughness of 7.8 μm. However, the corresponding surface exhibited the hydrophilicity with the contact angle of ~36$^{o}$ as demonstrated in **Figure S2**d. These results revealed that the superhydrophobicity of the interface strengthened PVDF membrane was endowed by FOTS modified $TiO_2$nanoparticles.

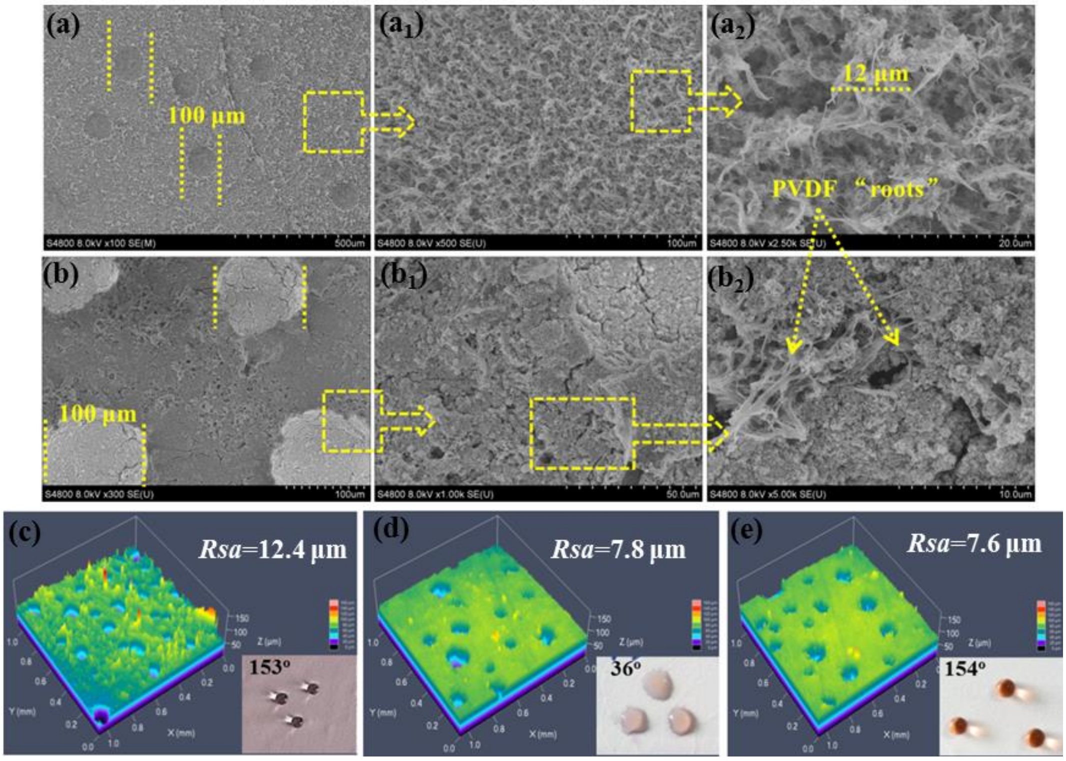


**Figure S2.**(a)-($a_2$) SEM images of the pristinePVDF membrane with different scales: 500 μm (a), 100 μm ($a_1$), 20 μm ($a_2$). (b)-($b_2$) SEM images of the rigid PVDF membrane with different scales: 100 μm (b), 50 μm ($a_1$), 10 μm ($a_2$). (c)-(e) 3D images of the soft PVDF membrane, PVDF membrane with the pristine $TiO_2$ coating, and the PVDF membrane with the FOTS-g-$TiO_2$ coating. The insert photographs are the water contact angle.

In **Figure** S3a and **Table S**1, the characteristic peaks at 1403, 1383, 1211, 1180, 1151, and 1068 cm$^{-1}$ attributing to the -CF- were observed for the pristine PVDF membrane. Correspondingly, the characteristic peaks at 686 eV and 285 eV with the mass percentage of 60.2 wt.% and 38.9 wt.% attributing to the F and C, respectively, were obtained for the special PVDF membrane. After coated by the $TiO_2$nanoparticles,

the surface chemical characteristic of PVDF membrane was changed. As observed in **Figure S**3b and **Table S**1, the intensity of -CF- originated from PVDF membrane was reduced with the pristine $TiO_2$ coating, and the mass percentage of F and C was significantly decreased, and the new peaks at 456 eV and 530 eV attributing to the Ti and O distinctly appeared, indicating the PVDF membrane surface was mainly fabricated by the pristine $TiO_2$ coating. Furthermore, as seen in **Figure S**3c and **Table S**1, the intensity of -CF- of the PVDF membrane was increased with the FOTS-g-$TiO_2$ coating in comparison with that of the pristine $TiO_2$ coating, and the mass percentage of F was significantly increased from 9.9 to 33.0 wt.%, and the new peak at 150 eV and 100 eV attributing to the Si of FOTS appeared, indicating the existence of FOTS on the $TiO_2$ coating of PVDF membrane surface.

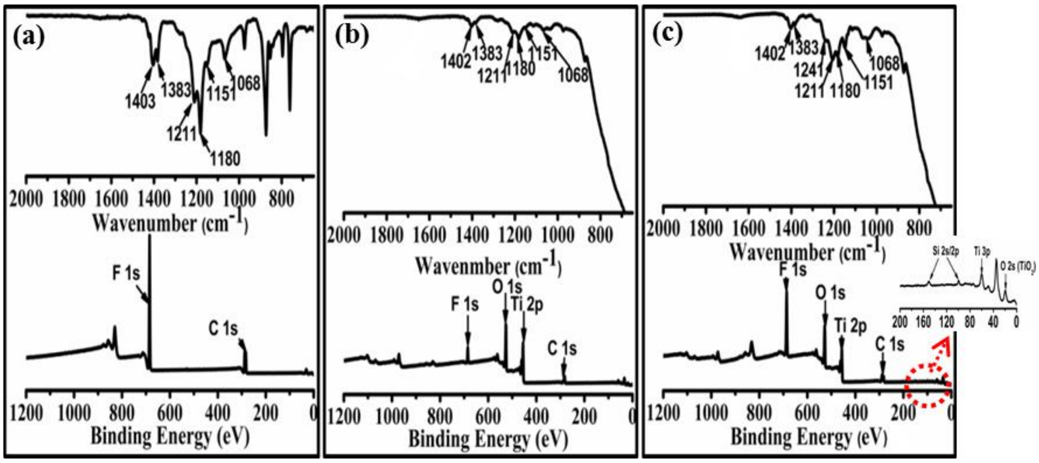


**Figure S3.** (a)-(c) The FTIR and XPS spectra of the pristine PVDF membrane (a), PVDF membrane manipulated by the pristine $TiO_2$ nanoparticles (b), PVDF membrane manipulated by the FOTS-g-$TiO_2$ nanoparticles (c), respectively. The insert picture is the magnified XPS spectra of the PVDF membrane strengthened by the FOTS-g-$TiO_2$ nanoparticles.

**TableS1.** Elemental mass percentage obtained from the XPS

| Sample | Percentage of components (wt.%) | | | | |
|---|---|---|---|---|---|
| | **F** | **O** | **Ti** | **C** | **Si** |
| a | 60.2 | 0.9 | 0 | 38.9 | 0 |
| b | 9.9 | 32.6 | 42.0 | 15.5 | 0 |
| c | 33.0 | 21.5 | 27.8 | 14.6 | 3.1 |

Note: sample a is the pristine PVDF membrane, sample b is the rigid PVDF membrane decorated by the pristine $TiO_2$, sample c is the rigid PVDF membrane manipulated by the TOFS-g-$TiO_2$.

On the other side, the delicate polymeric microstructures are usually subject to the thermal deformation due to the inherent flexibility. Given this, the thermal resistant testing was performed with increasing temperature under 10 MPa. As shown in **Figure S4** and **Movie S**7, the WCA of the soft superhydrophobicPVDF membrane was reduced to 100$^o$ under 10 MPa loading pressure at room temperature. With the temperature increasing to 80 $^o$C, WCA was further decreased to about 80$^o$, demonstrating the brittleness of of polymeric micro-/nano-structures. In comparison, the WCA of the rigid superhydrophobicPVDF membrane remained around 150$^o$ without losing its superhydrophobicity when the temperature is up to 180 $^o$C near the melting temperature of PVDF. Overall, as discussed above, the perfluorosilane$TiO_2$ interface provided the flexible PVDF membrane with robust superhydrophobic surface withstanding the extremely physical attack.

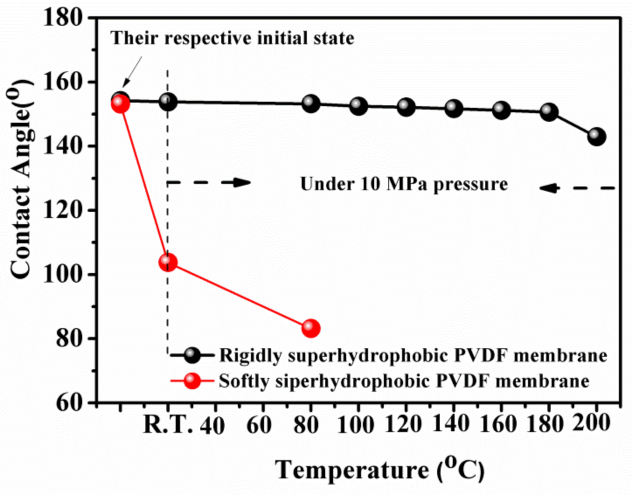

**Figure S** 4. Contact angle variation of the pristine soft PVDF membrane surfaces and rigid PVDF membrane surface manipulated by perfluorosilane$TiO_2$ coating after thermal and pressure tests.

**The Separation Efficiency of Water-in-Oil Emulsions: Figure S**5 shows optical microscopy images and size distribution of various water-in-oil emulsions as examples. It is observed that the emulsifier stabilized emulsions are non-transparent and milky, which was caused by the numerous dispersed nano-/micro- droplets. The average size of emulsions is ~4.69 μm, ~1.48 μm, ~2.09 μm, ~5.77 μm, and ~1.90 μm, respectively, for those water-in-oil emulsions involving in toluene, chloroform, n-hexane, soybean oil, and paraffin oil, as exhibited in **Table S**2. The average sizes of the emulsions were calculated by the software Image-ProPlus 6.0 from the optical microscopy images in **Figure S**5. After the dead-end filtration, not a single droplet is observed in the five collected filtrates in the whole optical microscopy images and the five filtrates are all transparent, indicating the effectiveness of the rigid superhydrophobicPVDF membrane for separating various water-in-oil emulsions.

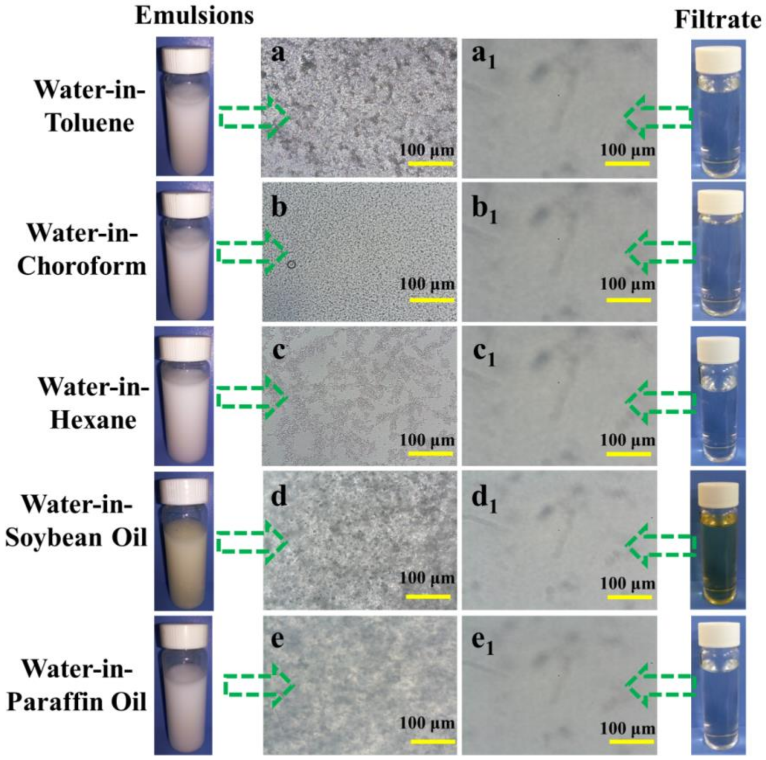


**Figure S 5.** Optical observation of various water-in-oil emulsions after dead-end separation: (a, $a_1$) $H_2O$-in-Toluene emulsion, (b, $b_1$) $H_2O$-in-Chloroform emulsion, (c, $c_1$) $H_2O$-in-Hexane emulsion, (d, $d_1$) $H_2O$-in-Soybean Oil emulsion, (e, $e_1$) $H_2O$-in-Paraffin Oil.

**Table S2.** Nano-/micro- size distribution and viscosity of the five emulsions

| **Emulsion** | **$H_2O$-in-Toluene** | **$H_2O$-in-Chloroform** | **$H_2O$-in-Hexane** | **$H_2O$-in-Soybean Oil** | **$H_2O$-in-Paraffin Oil** |
|---|---|---|---|---|---|
| Average Size Distribution (μm) | 4.69 | 1.48 | 2.09 | 5.77 | 1.90 |
| Viscosity (mPa.s) | 0.571 | 0.647 | 0.332 | 50.09 | 31.36 |
